# Springing into action: Comparing escape responses between bipedal and quadrupedal rodents

**DOI:** 10.1002/ece3.70292

**Published:** 2024-09-20

**Authors:** Grace A. Freymiller, Malachi D. Whitford, Craig P. McGowan, Timothy E. Higham, Rulon W. Clark

**Affiliations:** ^1^ Department of Biology Clovis Community College Fresno California USA; ^2^ Department of Biology San Diego State University San Diego California USA; ^3^ Department of Evolution, Ecology, and Organismal Biology University of California, Riverside Riverside California USA; ^4^ Keck School of Medicine, University of Southern California Los Angeles California USA; ^5^ Chiricahua Desert Museum Rodeo New Mexico USA

**Keywords:** antipredator, ground squirrel, kangaroo rat, pocket mouse, predator–prey, woodrat

## Abstract

Predation is a fundamental selective pressure on animal morphology, as morphology is directly linked with physical performance and evasion. Bipedal heteromyid rodents, which are characterized by unique morphological traits such as enlarged hindlimbs, appear to be more successful than sympatric quadrupedal rodents at escaping predators such as snakes and owls, but no studies have directly compared the escape performance of bipedal and quadrupedal rodents. We used simulated predator attacks to compare the evasive jumping ability of bipedal kangaroo rats (*Dipodomys*) to that of three quadrupedal rodent groups—pocket mice (*Chaetodipus*), woodrats (*Neotoma*), and ground squirrels (*Otospermophilus*). Jumping performance of pocket mice was remarkably similar to that of kangaroo rats, which may be driven by their shared anatomical features (such as enlarged hindlimb muscles) and facilitated by their relatively small body size. Woodrats and ground squirrels, in contrast, almost never jumped as a startle response, and they took longer to perform evasive escape maneuvers than the heteromyid species (kangaroo rats and pocket mice). Among the heteromyids, take‐off velocity was the only jump performance metric that differed significantly between species. These results support the idea that bipedal body plans facilitate vertical leaping in larger‐bodied rodents as a means of predator escape and that vertical leaping likely translates to better evasion success.

## Graphical abstract

The findings of this study suggest that enlarged hindlimbs improve escape performance by allowing rodents to more efficiently utilize vertical jumping as an antipredator escape response.

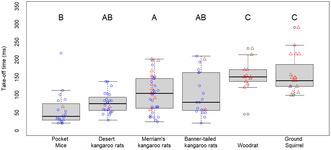

## 1 INTRODUCTION

Predators have evolved immensely diverse hunting strategies, ranging from active pursuit to cryptic, sit‐and‐wait ambush strategies. Therefore, in any ecosystem, prey must balance and contend with predation pressure coming from multiple predators with varied hunting strategies. The optimal escape strategy for prey to maximize their chance of evasion is often directly based on the predator's hunting strategy, and this has the potential to place strong selective pressure on the morphology of an animal (Arnold, 1983; Garland Jr. & Losos, 1994; Jayne & Bennett, 1990; Strobbe et al., 2009). For example, *Daphnia* exhibit different induced morphological defenses when faced with different predators, and the morphology that is induced by one predator may even reduce the *Daphnia* escape ability when faced with a different predator (Herzog & Laforsch, 2013). Thus, animals may evolve unique morphological attributes that support escape behaviors that are particularly effective against attack strategies of their primary predators in the environment.

In the deserts of North America, rattlesnakes and owls are common and abundant predators of rodents. Of the various rodent species that live alongside these predators, bipedal kangaroo rats are found in owl pellets less frequently than expected based on population densities (Kotler, 1985), and they are less likely than some quadrupedal rodents to be captured by owls (Longland & Price, 1991) and rattlesnakes (Pierce et al., 1992; Whitford et al., 2019) when attacked. Bipedalism is a relatively uncommon mode of locomotion for mammals, and it is accompanied by specialized morphological features such as enlarged hind limbs and reduced forelimbs. Elongated hindlimbs may confer a variety of advantages for rodents, one of which is improved jumping performance (Bradley‐Cronkwright et al., 2024). Within Rodentia, bipedality appears to have originated in ancestral species that occupied forested environments prior to their adaptive radiations into open, arid environments, possibly as an adaptation for vertical jumping to avoid predators in dense habitats (McGowan & Collins, 2018; Voorhies, 1975; Wu et al., 2014). Both rattlesnakes and owls are single‐strike predators, meaning if prey escape the initial attack, the predator is unlikely to capture it in a subsequent attempt (Kardong & Bels, 1998; Shifferman & Eilam, 2004). Because these attacks occur rapidly and on relatively small spatial scales, kangaroo rats rely on rapid vertical leaps to evade both snakes and owls (Freymiller et al., 2019; Higham et al., 2017; Webster, 1962; Whitford et al., 2019). These observations support the hypothesis that bipedal rodents are more effective at evading predators due to their morphological specialization, but a few studies have directly compared the mechanics of the evasive maneuvers of bipedal and quadrupedal rodents.

To date, studies that directly compare the escape kinematics and performance of bipedal to quadrupedal rodents have focused solely on running ability. Jerboas and kangaroo rats utilize zig‐zagging patterns when running away from a simulated predator attack, making their escapes more erratic and less predictable (Djawdan, 1993; Djawdan & Garland Jr., 1988; Moore et al., 2017). Additionally, compared to quadrupedal rodents, kangaroo rats reach higher maximum speeds (Djawdan & Garland Jr., 1988) and have higher running endurance (Djawdan, 1993). While these differences in running ability are important for escaping cursorial predators such as coyotes and foxes, they do not fully explain the lower predation rate by ambush, single‐strike predators (i.e., owls and snakes) (Kotler, 1985; Longland & Price, 1991; Pierce et al., 1992). Thus, there is a need for direct comparisons of variation in jump ability between bipedal and quadrupedal rodents.

We compared the evasive jumping ability of kangaroo rats (*Dipodomys* spp.), bipedal rodents common throughout North America, to that of three sympatric, quadrupedal rodents: pocket mice (*Chaetodipus* spp.), woodrats (*Neotoma* spp.), and ground squirrels (*Otospermophilus* spp.) (Brehme et al., 2011). All of these rodents are common prey of ambush‐hunting rattlesnakes (*Crotalus* spp.), one of the most abundant predators of small mammals in arid environments (Beavers, 1976; Clark et al., 2012; Cochran et al., 2021; Macartney, 1989; Nowak et al., 2008; Putman et al., 2016; Taylor, 2001). Kangaroo rats are well known for their impressive evasive antipredator leaps, which have been the focus of several recent kinematic studies (Freymiller et al., 2019; Higham et al., 2017; Schwaner et al., 2018, 2021; Whitford et al., 2019). Pocket mice are quadrupedal, but as heteromyid rodents, they share some common anatomical features with kangaroo rats, such as enlarged hindlimb muscles, reduced forelimbs, and enlarged auditory bullae (Bartholomew Jr. & Cary, 1954; Hatt, 1932; Webster & Webster, 1980). However, their evasive jumping ability has never been studied experimentally (but see Bartholomew Jr. & Cary, 1954, for qualitative descriptions). Given that pocket mice are morphologically similar and share a relatively recent common ancestor with kangaroo rats (Alexander & Riddle, 2005; Hafner et al., 2007), we also included woodrats and ground squirrels to incorporate a more diverse array of species (Blanga‐Kanfi et al., 2009). Woodrats have also never been studied in terms of evasive jumping abilities, and to our knowledge, the evasive jumping of ground squirrels has been examined in only one instance (Putman & Clark, 2015) with no direct comparisons to bipedal rodents.

We predicted that kangaroo rats, when compared to quadrupedal rodents that are also preyed on by rattlesnakes, would execute faster, more vertical jumps away from a simulated snake strike, and that pocket mice would perform best among the quadrupedal rodents given their anatomical similarity to kangaroo rats. Based on previous research demonstrating that heteromyids have enlarged auditory bullae facilitating rapid detection of auditory cues from predator attacks (Webster, 1962; Webster & Webster, 1971), we also predicted that heteromyids would have faster reaction times compared to non‐heteromyid rodents.

## 2 MATERIALS AND METHODS

### 2.1 Study sites and animals

All methods were approved by the San Diego State University Institutional Animal Care and Use Committee [APF 16‐08‐014C]. We targeted three species of kangaroo rat: the desert kangaroo rat (*Dipodomys deserti*, Stephens), Merriam's kangaroo rat (*Dipodomys merriami*, Mearns), and banner‐tailed kangaroo rat (*Dipodomys spectabilis*, Merriam). These species were chosen as they encompass the relatively large variation in body size seen among kangaroo rats. Additionally, data were collected for the desert pocket mouse (*Chaetodipus penicillatus*, Woodhouse) and the white‐throated woodrat (*Neotoma albigula*, Hartley). Data for California ground squirrels (*Otospermophilus beecheyi*, Richardson) were obtained from a previous study from our research group examining the effect of vigilance on squirrel escape responses using a similar methodology (detailed below).

Our study took place at several sites throughout southwestern North America. Initial data were collected from mid‐May through early August in 2016 at a site located within the Barry M. Goldwater Range outside of Yuma, Arizona, USA (*n* = 5 desert kangaroo rats). In mid‐May through early August of 2018, we collected additional data at a site in Rodeo, New Mexico, USA (31 banner‐tailed kangaroo rats, 25 Merriam's kangaroo rats, and 12 pocket mice) and at a nearby site in Animas, New Mexico, USA (15 banner‐tailed kangaroo rats). We collected data from June to July 2019 in the Mojave Desert of California at a site south of the California State University's Desert Studies Center located in Zzyzx, California, USA (22 desert kangaroo rats and 22 Merriam's kangaroo rats). Lastly, we revisited the Rodeo site from mid‐June to early August in 2020 (1 Merriam's kangaroo rat, 10 pocket mice, and 14 woodrats).

Rodents were trapped using Sherman live traps baited with heat‐sterilized black oil sunflower seeds. Traps were set between sunset and sunrise near burrows or middens. Trapped individuals were sexed and measured (mass, snout‐anus length, tail length, and hind foot length), then marked with fingerling ear tags (National Band and Tag #1005‐1) for long‐term identification and a unique fur dye mark using Nyanzol dye for short‐term identification. Fur dye marks ensured that rodents could be reliably identified during experiments and to prevent retesting of individuals. Individuals were measured and marked in the field and immediately released at the site of capture. Average body mass for each species is reported in Table 2.

### 2.2 Experimental procedure

We used a modified version of the methodology detailed in Freymiller et al. (2017) and Putman and Clark (2015) to record rodent evasive leaps. Once a marked rodent was reliably relocated (i.e., home burrow identified or an individual was found in the same area at least twice via traps and/or visual surveys), an experimental setup was placed in the vicinity of the known individual's location. The setup consisted of a rattlesnake strike simulator (RSS), infrared lighting (850 nm wavelength), and a GoPro video camera (Hero 4 Black) retrofitted with an IR‐sensitive lens (Peau Productions, 2.97 mm f/4.0 90d HFOV 5MP, no IR filter) recording at 240 frames per second (fps). A second video camera (Sony Handycams, model SR‐65 or SR‐300) recording at 30 fps was used to record the entirety of the trial and observe the animal during baseline feeding but was not used to collect videos for analysis. The RSS consists of a one‐inch diameter PVC pipe housing and a compressed spring that projects a cork toward a target at 2.8 m s^−1^, approximately the same velocity as a rattlesnake strike (Higham et al., 2017; Penning et al., 2016; Whitford et al., 2019). To hold the spring while compressed until the trial was ready to begin, we attached a piece of monofilament nylon line to the end of the spring, then tied the other end to a camera tripod manned by an observer 3–5 m away.

At the beginning of a trial, the target individual was allowed to approach and inspect the RSS. They were encouraged to feed near the device by baiting it with sunflower seed. Most rodents did not appear disturbed by the presence of the RSS, but the trial was immediately ended if an individual behaved apprehensively (e.g., through antipredator displays), as vigilance/alertness can affect jump performance (Freymiller et al., 2017; Putman & Clark, 2015). Examples of behaviors that were considered vigilance behaviors varied depending on the species but included behaviors such as foot drumming, sand kicking, jump backs, and frequent head‐up scanning sustained for at least 1 s (Freymiller et al., 2017). When an individual approached the seed pile, the monofilament line was cut which released the spring and cork, and the rodent's response was recorded. If the rodent jumped and remained in frame (Table 1, Video 1), the horizontal displacement, defined as the distance (in m) between the take‐off and landing positions, was immediately measured in the field with a tape measure using the video playback for guidance. If the rodent did not jump, the trial was classified as a “scramble” (Table 1, Video 2) and horizontal displacement was not measured. These trials were retained for reaction time and take‐off time calculations but were not used in performance analyses. Individuals were only tested once to prevent the possibility that learning would affect the response to the RSS. All trials were recorded between sunset and sunrise. As light levels at night vary widely based on the moon phase and could affect the rodents' ability to see the cork (and therefore influence reaction time), ambient light was measured with a digital light meter (Extech LT300) immediately after every trial.

**TABLE 1:** Video examples of rodent escape maneuvers.

Description	Video link
Video 1: examples of rodent jumps	https://youtu.be/j38‐ZygU7MM
Video 2: examples of rodent scrambles	https://youtu.be/BZTaqe1G1PQ
Video 3: examples of kangaroo rats and pocket mice escaping rattlesnake strikes (footage from Higham et al., 2017)	https://youtu.be/piNuJHAM8FU

In 2016, two paired high‐speed cameras (Edgertronic, model SC1) recording at 500 fps and connected to laptop computers via 100 ft Ethernet cables were used to record the evasive jumps instead of a single GoPro camera. These videos were calibrated with a large object of known dimensions (metal rods fixed to a 30 × 25 cm metal plate) for three‐dimensional analyses. To make these videos comparable to the GoPro recordings, the frame rate was reduced by converting the videos to a series of still images using the “magick” package (Ooms, 2021) in R (version 4.0.3) and down‐sampled to 250 fps. Using the three‐dimensional calibration, the horizontal displacement of the jump was extracted by digitizing a point on the toes in the frame of toe‐off and in the frame of landing using the software DltDataviewer, version 7 (Hedrick, 2008) in MATLAB (R2018b). This horizontal displacement was then used in the jump performance calculations in the same way that we used the displacement values measured in the field for the other trials.

### 2.3 Incorporation of data from previous studies

In order to allow for a broader comparison of rodent performance, we incorporated data from Putman and Clark (2015). California ground squirrels were tested with the RSS at a site approximately 20 miles east of San Jose, CA, USA, from May–August in 2012 and 2013 (*n* = 23 ground squirrels). The methods between this study and the present study differ in several ways. First, horizontal displacement was not measured for ground squirrel trials, so we could only use these data for the reaction time and take‐off time analyses. Second, the rodents' responses were filmed at 120 fps instead of 240–250 fps, so the measurements of reaction time and take‐off time are at a coarser timescale than the measurements for the other five species (i.e., each frame is 8.3 ms in the squirrel videos and 4–4.2 ms in the other videos). However, as the error rate in these values is ±2 frames, this discrepancy is only relevant for measurement differences less than 5 frames. Third, the length of the device in the present study was about half as long as the one used for the ground squirrels. However, because the cork was always tied back so that it aligned with the edge of the PVC pipe in both studies, and because the velocity of the devices was very similar (3.1 and 2.8 m s^−1^), we do not expect this difference to affect the analyses. Lastly, because ground squirrels are strictly diurnal, all ground squirrel trials were recorded during the day. While this could impact factors such as reaction time, daytime trials are the most ecologically relevant (and feasible) for ground squirrels, as they are not active outside of burrows at night.

### 2.4 Video and statistical analyses

We used the GoPro video recordings to quantify several variables associated with the rodents' evasive maneuvers, including reaction time, take‐off time, take‐off velocity and angle, and jump height. Reaction time was measured as the time between the first movement of the cork and the first visible movement of the rodent's reaction. If the rodent did not react until after the cork made contact with the individual (i.e., they were hit with the cork, *n* = 20 trials) or they seemed to react before the cork started to move (*n* = 2 trials), the reaction time was not measured. Take‐off time was measured as the time from the first visible movement of the rodent's reaction to the frame immediately preceding toe‐off. For scramble maneuvers, toe‐off was defined as the last frame in which the hind feet were on the ground (i.e., the frame immediately before the animal propelled its body away from the simulated attack path). Thus, take‐off time does not include reaction time but rather is a measurement of how quickly the animal can move its body from the path of the cork once it starts to react.

Using the horizontal displacement measured in the field and the amount of time spent airborne (measured as the number of seconds between the take‐off and landing frames), we calculated take‐off velocity (m s^−1^), take‐off angle (°), and jump height (m) using the following standard ballistic equations (as in Freymiller et al., 2017):

(1) $${\text{Velocity}}_{h}=\frac{\text{horizontal displacement}}{\text{time spent in}\;\mathrm{air}\;}$$

(2) $${\text{Velocity}}_{v}=g\left(\frac{\text{time spent in}\;\mathrm{air}\;\left(s\right)}{2}\right)$$

(3) $$\text{Take}‐\mathrm{off}\;\text{velocity}=\sqrt{{\text{Velocity}}_{h}^{2}+{\text{Velocity}}_{v}^{2}}$$

(4) $$\text{Jump angle}=\text{atan}\left(\frac{{\text{Velocity}}_{v}}{{\text{Velocity}}_{h}}\right)\times \frac{180}{\pi }$$

(5) $$\text{Jump}\;\text{height}=\frac{{\text{Velocity}}_{v}^{2}}{2\times g}$$

where *g* = acceleration due to gravity (9.8 m s^−2^). The jump height data collected from the three kangaroo rat species (as well detailed hind limb morphology data) have been previously published in an earlier manuscript examining the scaling of jump performance and hind limb morphology (Freymiller et al., 2021).

All statistical analyses were performed in R (version 4.0.3). We analyzed species differences in jump performance using a permutational multivariate analysis of variance (PERMANOVA) from the R package “vegan” (Oksanen et al., 2019). First, we corrected take‐off time, take‐off angle, and jump height for body size by regressing the log of each variable against the log of body mass for the pooled species. Individuals without a body size measurement were excluded from this analysis. We then used the residuals from each body mass regression as the dependent variables (collectively termed “overall performance”) for the multivariate analysis, and species as the independent variable. The other relative body measurements (hindfoot length, tail length, and snout‐anus length) were not included in the analyses due to the challenges of measuring these features with enough precision on unanesthetized animals in the field. We used Euclidean distance on unit‐standardized performance variables and 999 permutations.

Reaction time and take‐off time were each analyzed with separate linear models with species as the only independent variable, and a Tukey's HSD test was used for pairwise species comparisons. Raw data for reaction time and take‐off time (i.e., not standardized for body size) were used in the analyses. We were also interested in the relationship between escape mode (jumping/scrambling) and reaction time and take‐off time. However, only Merriam's kangaroo rats had a large enough sample size of both jumps and scrambles, so they were the only species included in those analyses. We explored the relationship between reaction time and the probability a rodent would jump using a logistic regression, and we used a linear model to compare take‐off times between individuals within a species, which scrambled and those which jumped. We excluded ambient light in the model of reaction time as there was little variation in our recorded values (our light meter had a minimum sensitivity of 0.01 lux, and only 20% of trials occurred at levels above 0.02 lux). As head position (up/down), distance from the RSS, and body mass were not significant factors affecting reaction time or performance in an earlier study of kangaroo rat escape maneuvers (Freymiller et al., 2017), we did not include those metrics in our analyses. Phylogenetic comparative methods were not utilized in any of the analyses due to the small number of species in the study (Garland Jr. & Adolph, 1994). Data are available as a supplementary document (Data S1).

## 3 RESULTS

### 3.1 Sample sizes and jump frequency

We collected data for a total of 48 Merriam's kangaroo rats, 46 banner‐tailed kangaroo rats, 27 desert kangaroo rats, 22 desert pocket mice, and 14 white‐throated woodrats, and data were incorporated for 23 California ground squirrels. The large difference in overall samples for each species is due to differences in the propensity to jump; species that jump less frequently required more trials to reach an adequate sample size of jumps. Merriam's kangaroo rats jumped 63% of the time (30/48 trials), and banner‐tailed kangaroo rats jumped 74% of the time (34/46). Comparatively, desert kangaroo rats jumped 93% of the time (25/27) and desert pocket mice jumped 91% of the time (20/22). Ground squirrels only jumped in 9% of trials (2/23), and woodrats only jumped 7% of the time (1/14), so it was not feasible to gather a large enough sample of jumps for either of these species. Because we could not always collect each variable of interest from every trial due to variations in video quality or rodent behavior (e.g., if the rodent was struck with the cork, we could not measure reaction time, but we could measure jump performance variables), we report the final sample sizes for each analysis along with the means for each measured variable in Table 2.

**TABLE 2:** Summary statistics of jump variables and sample sizes (*n*) for each species (DIME = Merriam's kangaroo rat, DIDE = desert kangaroo rats, DISP = banner‐tailed kangaroo rats, CHPE = desert pocket mice, NEAL = white‐throated woodrats, OTBE = California ground squirrels).

	Mass (g)	Reaction time (ms)	Take‐off time (ms)	Overall response time (ms)	Take‐off velocity (m s^−1^)	Jump height (cm)	Take‐off angle (°)
DIME	40 ± 1	22.8 ± 1.2 (*n* = 39)	103.1 ± 8.3 (*n* = 38)	126.4 ± 9.4 (*n* = 38)	2.1 ± 0.1 (*n* = 29)	8.6 ± 1.5 (*n* = 29)	39.7 ± 3.4 (*n* = 29)
DIDE	89 ± 5	18.8 ± 1.5 (*n* = 24)	77.8 ± 6.0 (*n* = 24)	96.5 ± 7.0 (*n* = 24)	2.6 ± 0.1 (*n* = 25)	11.2 ± 1.8 (*n* = 25)	35.3 ± 3.8 (*n* = 25)
DISP	116 ± 3	29.4 ± 1.4 (*n* = 24)	101.9 ± 13.5 (*n* = 20)	131.5 ± 14.1 (*n* = 20)	2.4 ± 0.1 (*n* = 27)	7.2 ± 1.1 (*n* = 27)	31.6 ± 3.1 (*n* = 27)
CHPE	18 ± 1	28.4 ± 2.2 (*n* = 21)	58.2 ± 10.5 (*n* = 20)	86.1 ± 11.5 (*n* = 20)	2.2 ± 0.1 (*n* = 19)	9.2 ± 1.1 (*n* = 19)	39.2 ± 3.2 (*n* = 19)
NEAL	177 ± 21	19.1 ± 1.0 (*n* = 13)	152.5 ± 12.3 (*n* = 13)	171.6 ± 11.9 (*n* = 13)	–	–	–
OTBE	523 ± 37	36.8 ± 2.1 (*n* = 21)	157.7 ± 12.2 (*n* = 19)	186.8 ± 10.0 (*n* = 19)	–	–	–

*Note*: Woodrats and ground squirrels did not have jump kinematic metrics because they rarely jumped. Overall response time was calculated for each individual as the sum of reaction time and take‐off time. Values are mean ± standard error.

### 3.2 Jump performance

**FIGURE 1:**
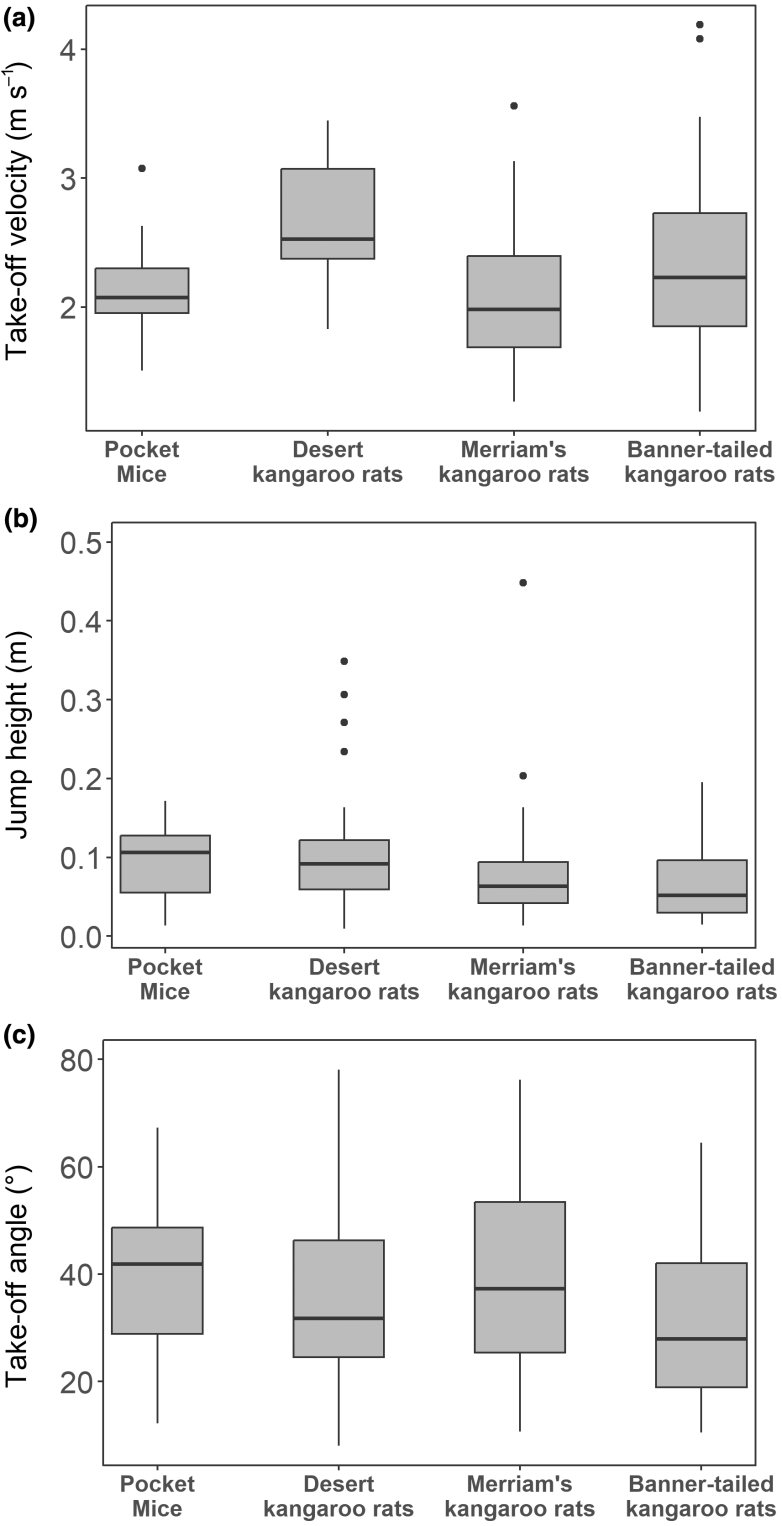
Jump performance comparisons among heteromyid rodents in take‐off velocity (a), jump height (b), and take‐off angle (c). Species showed no significant differences in velocity, and all species made low, relatively horizontal jumps.

Trials in which individuals scrambled, jumped off‐screen, or the video quality was too poor to extract the necessary information were removed from these analyses. Woodrats were completely excluded because only one individual jumped and it landed off‐screen, preventing statistical analyses. Ground squirrels were also excluded because only two individuals jumped, and the horizontal displacement values were not measured in that dataset so performance metrics could not be calculated. Overall jump performance was not significantly different among the species retained (*F*_3,96_ = 1.4, *p* = .2; Figure 1). After correcting for body size, heteromyid species (including the quadrupedal pocket mouse) performed similarly during jump escapes.

### 3.3 Reaction time and take‐off time

**FIGURE 2:**
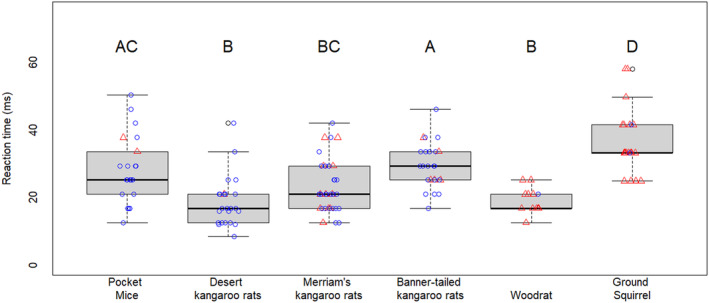
Comparison of reaction time among rodent species. Diurnal ground squirrels had the overall slowest reaction time compared to the other species, which are all nocturnal. Average woodrat reaction time was faster than many of the heteromyid species. Letters show significant differences according to a Tukey's HSD post hoc test. Individuals that scrambled are represented with red triangles. Individuals that jumped are represented with blue circles.

We removed trials in which (1) the individual was hit with the cork before initiating a reaction, (2) the animal began an evasive maneuver before the cork began to move, or (3) the video quality was not sufficient to see the first movement of the animal. We removed individuals that were hit with the cork before initiating a response as those measurements would not be comparable to the others: any measurement of reaction time in those instances could be a measurement of how long it took the individual to react from being physically struck, rather than reaction time to an oncoming simulated predator attack. Based on the logistic regression with Merriam's kangaroo rats, there was no relationship between the probability of jumping and reaction time (mean scramble reaction time 26 ms, mean jump reaction time 24 ms; odds ratio = 1.00, *p* = .97). Therefore, we included all trials (i.e., both scrambles and jumps) for all species in the final model. There were significant differences among species in reaction time: desert kangaroo rats and woodrats reacted faster than the other species, and ground squirrels reacted significantly slower than all the other species (*F*_5,136_ = 16.1, *p* < .001; Figure 2). Merriam's kangaroo rats, pocket mice, and banner‐tailed kangaroo rats all had intermediate reaction times.

**FIGURE 3:**
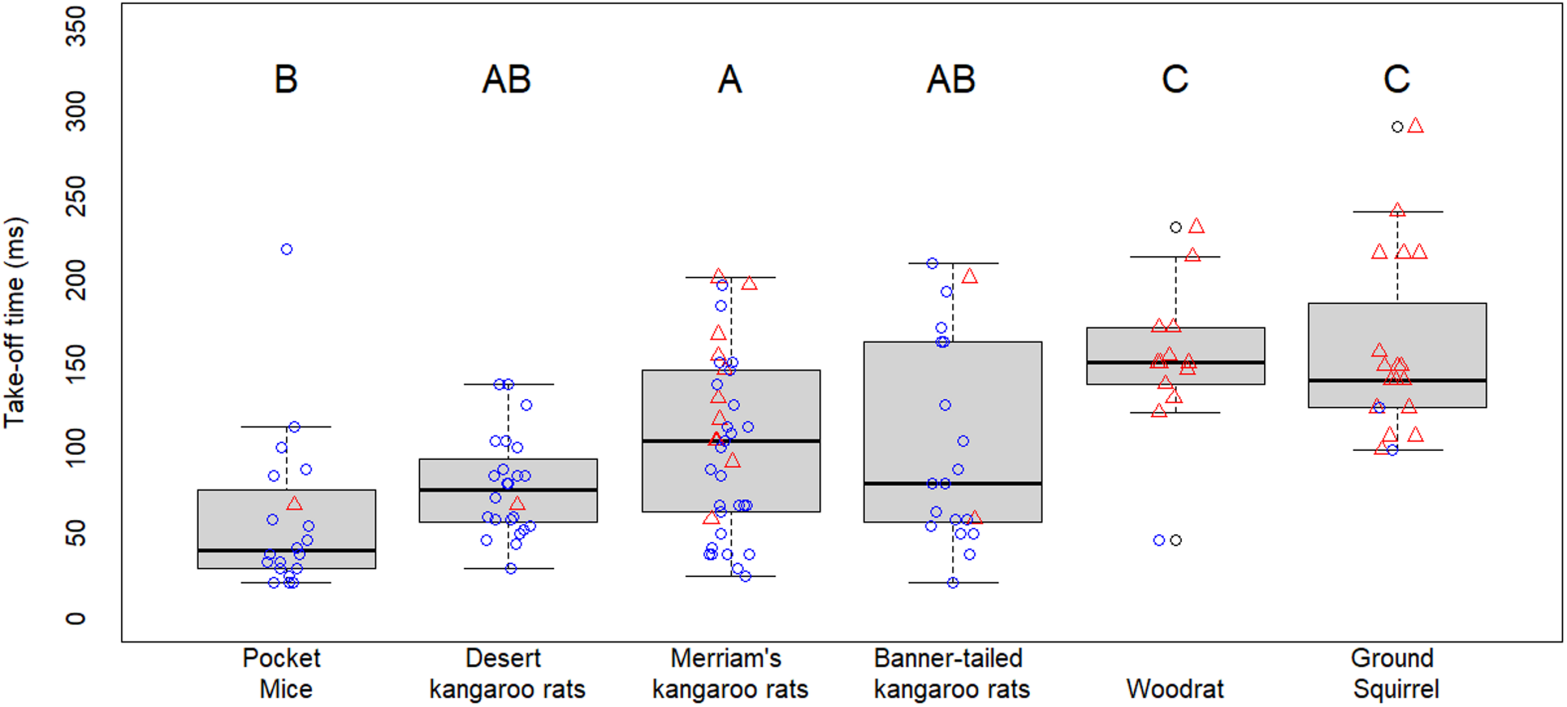
Comparison of take‐off time among rodent species. Non‐heteromyid rodent species had significantly longer take‐off times than heteromyid species, largely due to their propensity to scramble rather than jump and therefore need to reorient and turn before escaping (ground squirrels and woodrats jumped in less than 10% of trials, compared to the kangaroo rats and pocket mice, which jumped in over 60% of the trials). Letters show significant differences according to a Tukey's HSD post hoc test. Individuals that scrambled are represented with red triangles. Individuals that jumped are represented with blue circles.

For the take‐off time analyses, we also had to remove trials in which the video quality was not sufficient to determine the frame of toe‐off and/or see the first movement of the animal. Pocket mice had the shortest average take‐off time, and non‐heteromyid quadrupeds had significantly longer take‐off times than the heteromyid rodents (*F*_5,128_ = 12.2, *p* < .001; Figure 3). The heteromyids had generally similar take‐off times, with only pocket mice and Merriam's kangaroo rats exhibiting significantly different mean values.

Merriam's kangaroo rats had significantly longer take‐off times when they scrambled compared to when they jumped (mean scramble take‐off time 126 ms, mean jump take‐off time 88 ms; *F*_1,36_ = 6.7, *p* = .01). Therefore, jump maneuvers produce a significantly faster escape from the attack trajectory. This is supported by the finding that woodrats and ground squirrels (which almost never jumped) had the overall slowest take‐off time, and the two kangaroo rat species which were most likely to scramble rather than jump (Merriam's and banner‐tailed kangaroo rats), had the slowest average take‐off times among heteromyids (Figure 3). In the few instances in which ground squirrels and woodrats jumped (2 times and 1 time, respectively), their take‐off times were comparable to the heteromyids (Figure 3).

## 4 DISCUSSION

The rodents in this study had highly variable startle responses and escape abilities. Only the heteromyid rodents were prone to jumping when startled, so we were unable to compare the evasive jump kinematics among all the rodents we tested. That said, the observed differences in jump probability and take‐off time may help explain why heteromyids are more successful in evading single‐strike predators (Kotler, 1985; Longland & Price, 1991; Pierce et al., 1992). Overall, heteromyids were much more likely to rely on jumping as a startle response to a high‐speed “attack” from our strike simulator than non‐heteromyids. This propensity to jump seems to translate to a more effective escape from ambush predators, as both quadrupedal and bipedal heteromyid rodents are less likely than non‐heteromyid rodents to be captured when attacked by owls (Kotler, 1985; Kotler et al., 1988; Longland & Price, 1991). The heavy reliance of heteromyids on jumping as a general escape maneuver, regardless of whether they are bipedal or quadrupedal, is likely driven by a combination of limb morphology, body posture, and size.

When jumping, animals must align their center of mass over the line of action of the propulsive force (i.e., over the hindlimbs) to avoid excessive angular momentum on the body as it moves through the air. The bipedal posture of kangaroo rats, as well as their elongated tails, keeps their center of mass near their hindlimbs during normal locomotion, allowing them to rapidly and smoothly execute a jump maneuver. Quadrupedal rodents, on the other hand, must first use their forelimbs to pitch their bodies over the hindlimbs before propelling themselves into the air. Both the pitching maneuver and the acceleration of the body takes less time and effort for a smaller‐bodied quadruped, such as a pocket mouse, which may explain why they were more likely to jump than woodrats and ground squirrels. Furthermore, the larger hindlimb musculature (relative to body size) of pocket mice compared to woodrats and ground squirrels likely shifts their center of mass closer to the hindlimbs, thus facilitating a faster jump maneuver. There may also be a link to the specialized hearing of heteromyids, as both pocket mice and kangaroo rats have enlarged auditory bullae that improve their sensitivity to low‐frequency sounds (Webster & Webster, 1980). Auditory cues are processed relatively quickly by the central nervous system (Davis, 1984; Nicolas, 1997), so it is possible that enlarged hindlimb musculature coevolved with specialized hearing within the Heteromyidae family to produce a phenotype that is well‐suited for rapid escape jumps. While we do not have the spectrum of species needed to perform phylogenetically informed analyses, it is important to note that other bipedal species, such as jerboas and springhares, also have enlarged auditory bullae.

It is also possible that squirrels and woodrats were not as motivated to jump for reasons that do not relate directly to being quadrupedal. For example, perhaps these larger‐bodied mammals were not as threatened by the simulated attack and therefore not as motivated to jump. However, observations in other field‐based studies of rattlesnake hunting behavior also indicate a greater proclivity for evasive jumping by kangaroo rats. Of the four instances in which our research group has opportunistically recorded natural interactions between woodrats and rattlesnakes (situations in which motivation is presumably high), only one of those individuals jumped away from the snake, whereas kangaroo rats always exhibit repeated evasive jumps while investigating live snakes (Clark et al., 2016; Freymiller et al., 2017). Like woodrats, squirrels investigating live rattlesnakes only occasionally jump back from the snake (Ayon et al., 2017; Putman & Clark, 2015). However, we still recognize that a much broader comparative analysis of biped and quadruped species would be necessary to fully understand possible associations between bipedality and evasive jumping.

### 4.1 Take‐off time

When examining take‐off time, quadrupedal rodents outside of the heteromyid family take longer to move their bodies out of the path of an attack (Figure 3). This pattern is driven by the fact that the quadrupedal rodents need to turn and reorient their bodies before scrambling out of the trajectory of the RSS (Table 1, Video 2). The relationship between reorienting and extended take‐off time is even seen among the heteromyids: Merriam's kangaroo rats that scrambled had significantly slower take‐off times compared to Merriam's kangaroo rats that jumped because they took more time to orient their bodies onto an escape path. Furthermore, the two heteromyid species that were less likely to jump (Merriam's and banner‐tailed) had slower take‐off times than the two heteromyid species that had the highest propensity for jumping (desert kangaroo rats and desert pocket mice; Figure 3). In the few instances when the woodrats and ground squirrels did jump, their take‐off times were noticeably faster: the quickest take‐off time we recorded for woodrats was from the one individual that jumped (46 ms), and the next fastest take‐off time took almost three times as long (122 ms). If non‐heteromyid rodents jumped more frequently, it is likely that their average take‐off times would be much shorter.

It should be noted that woodrats and ground squirrels are capable of evasive jumping—we recorded one jump from a woodrat when it was physically struck by the cork (Table 1, Video 1), and ground squirrels jump more frequently when they are in a high‐vigilance state (Putman & Clark, 2015). However, unlike heteromyids, neither species appears to jump readily when in a “baseline” vigilance state. Given that both groups can jump, and jumping appears to be a more effective means of predator escape, why don't non‐heteromyid rodents rely more on jumping as a generalized escape response? Aside from the potential difficulty for these large‐bodied quadrupeds to adequately align their bodies quickly enough for a jump and subsequent body acceleration, there may be other factors at play. Injury risk could also contribute to the observed differences in jump probability, as kangaroo rat hindlimbs are well‐built for the rapid acceleration and force associated with their evasive leaps compared to more typical rodents (Biewener & Blickhan, 1988; Javidi et al., 2019; Rankin et al., 2018; Schwaner et al., 2018). Similarly, it is possible that venom resistance may drive differences in escape response, specifically to a simulated snake strike. Both ground squirrels and woodrats are known for their physiological resistance to rattlesnake venom (Biardi, 2008; Robinson et al., 2021), whereas none of the heteromyids used in this study are known to possess resistance (although a congener, *D*. *ordii*, appears to have evolved venom resistance in some parts of its range to some pit viper venoms; Balchan et al., 2024). Therefore, there might be an interaction between venom resistance and behavioral responses to rattlesnake stimuli among small mammals. Future studies could examine these possibilities experimentally by incorporating a wider variety of quadrupedal rodents and further quantifying venom resistance on heteromyid species.

Among heteromyids, the quadrupedal pocket mouse had the shortest average take‐off time. This could be explained simply by differences in overall body size as pocket mice are much smaller than kangaroo rats (Table 2). Smaller animals have shorter limbs, which reduces the amount of time they will be in contact with the ground during a jump. Therefore, the amount of time an animal takes to leave the ground during a jump is related to the absolute length of an animal's hindfoot (Hill, 1950).

As take‐off time in this study is a measure of how quickly an animal moves its body from the vector of an oncoming attack, it has important consequences for escape success. Scrambling adds significant time to the escape maneuver, which could be detrimental during a real rattlesnake or owl strike, as even small increases in take‐off time could give these high‐speed ambush predators the advantage. Rattlesnakes extend their coiled bodies quickly during predatory strikes; on average, they make contact/bite prey within approximately 135 ms (range of 54–308 ms; Whitford et al., 2019). Woodrats had an average take‐off time of 153 ms and ground squirrels had an average take‐off time of 158 ms, whereas the Merriam's kangaroo rats, which had the slowest take‐off time among heteromyids, had an average take‐off time of 103 ms (Table 2). Therefore, a kangaroo rat would be more likely to successfully escape when compared to their quadrupedal counterparts. The extra time that woodrats and ground squirrels take to evade could provide a rattlesnake with just enough time to close the gap between itself and its prey, highlighting the positive consequences of jumping on the likelihood of escaping the rapid attack of a single‐strike predator.

### 4.2 Jump performance

For the heteromyid species, there were no significant differences in regard to jump performance, and there does not appear to be a link between jumping ability and bipedality within this family. It should be noted that the body mass correction utilized in our statistical analyses likely reduced our ability to detect a species signal as species identity and body size are confounded; the average mass of the pocket mice in this study was approximately half that of the Merriam's kangaroo rat (the smallest kangaroo rat species in this study), and we did not have any similarly small bipedal species. Size correction is generally employed to remove differences in performance that may be attributed to overall body size rather than any specific morphological differences (e.g., do desert kangaroo rats jump the highest simply because they are a large species, or because of a fundamental difference in morphology/behavior/etc.). Comparing the species with respect to their raw (i.e., not size‐corrected) performance, as shown in Figure 1 and Table 2, there still appears to be little difference in jumping performance: on average, all species made low jumps that were more horizontal than vertical, and the most apparent differences were observed in take‐off velocity. While there were no extreme differences in performance detected in our study and no species was an outlier in any performance metric, there may be more nuanced differences that would require a wider array of species and a more detailed morphological assessment.

The similarity in jump performance of the quadrupedal desert pocket mouse and the bipedal kangaroo rat species demonstrates that at least some quadrupedal heteromyids are also capable of powerful evasive jumping. Pocket mice share many basic anatomical features with kangaroo rats, such as enlarged hindlimbs and reduced forelimbs, and have been considered a morphological intermediate between bipedal and quadrupedal forms (Bartholomew Jr. & Cary, 1954; Hatt, 1932). Indeed, pocket mice have been recorded escaping rattlesnake strikes with jumps that are qualitatively similar to those of kangaroo rats (Table 1, Video 3), but this may be a unique trait for a quadrupedal rodent that is driven by the pocket mouse's relatively recent shared evolutionary history with kangaroo rats (*Dipodomys* and *Chaetodipus* appear to have diverged approximately 22.3 million years ago; Hafner et al., 2007). Future studies incorporating larger quadrupedal heteromyids can shed more light on whether the lack of observed performance differences in this study can be attributed to morphological similarities between bipedal and quadrupedal heteromyids or an artifact of the study design.

One other important consideration is that the jumps recorded in this study were a general escape response and not necessarily reflective of the maximal performance of these animals. Animal performance varies widely both across and within various contexts, and performance in the wild is often not reflective of maximal performance as measured under artificial conditions (Hertz et al., 1988; Husak, 2006; Irschick & Garland Jr., 2001). As we only measured one escape per individual, we are not able to determine if the rodents in this study were performing maximally. Previous studies with desert kangaroo rats have found that they can jump almost a full meter into the air when alerted to the presence of a rattlesnake (Freymiller et al., 2017, 2019), whereas the average jump heights for the desert kangaroo rats in this study were a small fraction of that (often less than 10 cm). It is possible that the rodents did not perceive the RSS as a threat in the same way that they would a rattlesnake strike, leading to submaximal performance. Thus, species‐level differences in jump performance may become exaggerated when comparing maximal performance, which would help elucidate relationships between body size, bipedality, and jump performance.

### 4.3 Reaction time

In natural interactions between rattlesnakes and kangaroo rats, reaction time is one of the most important factors in determining if a kangaroo rat will successfully evade a strike (Whitford et al., 2019). Kangaroo rats are known for their enlarged auditory bullae and specializations to the middle ear bones, which help them detect low‐frequency sounds (Heffner & Masterton, 1980; Webster & Webster, 1980), such as those produced by a snake strike or owl swoop (Webster, 1962). Therefore, assuming that these nocturnal rodents are utilizing sound as a primary cue during the recorded evasions in this study, it is not surprising that kangaroo rats had extremely fast reaction times—in some instances as quick as 8–16 milliseconds. Bullar inflation is also observed in other heteromyid species, and larger bullar volumes are associated with increased use of open microhabitats, suggesting that the amplified risks of these habitats may be mitigated by improved hearing and detection of predators (Kotler, 1984). Pocket mice also show enlarged bullae compared to other small rodents, but not to the same extent as kangaroo rats, which may explain why their average reaction time was slower than some of the kangaroo rats.

The degree of variation among kangaroo rats (Figure 2) is unexpected and could be related to unknown anatomical or physiological differences. There does not appear to be any relationship to body mass as the relatively large desert kangaroo rat (average mass of 89 g in this study, Table 2) and the smaller Merriam's kangaroo rat (40 g), both had significantly faster reaction times than the banner‐tailed kangaroo rat (116 g). Among the 20 trials that we omitted because the individual was hit with the cork before initiating a reaction, 14 of those were banner‐tailed kangaroo rat trials. Given that the banner‐tailed kangaroo rats also had the slowest average reaction time, it is possible that some unknown difference in this species' morphology and/or physiology causes them to react more slowly than other *Dipodomys* species. For example, the relative size and scaling of auditory bullae among different kangaroo rat species has not been investigated. If some species have disproportionately small bullae (and less sensitive hearing), it could drive differences in reaction time that would not be apparent from external morphology.

Although woodrats are not known to have extremely sensitive hearing, they had significantly faster reaction times than ground squirrels (and actually faster than many of the heteromyids). This difference between ground squirrels and woodrats could be explained by the dominant sensory systems each species uses to detect predators. Woodrats are nocturnal and, like kangaroo rats, likely respond more strongly to auditory cues, whereas ground squirrels are diurnal and therefore likely rely more heavily on vision. Because auditory processing is more rapid than visual processing (Davis, 1984; Nicolas, 1997), enhanced sensitivity to auditory cues would be expected to result in a faster reaction time, even in the absence of specialized auditory morphology such as the enlarged bullae of heteromyids. Given the woodrats' fast reaction times, it is possible that this could offset the slower take‐off times associated with their scramble maneuvers exhibited in response to an ambush predator attack. However, even when considering both of these metrics together as a combined overall response time (Table 2), the fast woodrat reaction time still cannot adequately compensate for their slower take‐off time when considering the timing of a hypothetical rattlesnake strike, as discussed above. All four heteromyids have average response times under the average rattlesnake strike time (135 ms; Whitford et al., 2019), whereas both non‐heteromyid quadrupeds have average response times well above that average (Table 2).

We also caution that a more ecologically relevant interpretation of differences in reaction time among these nocturnal species would require a more ecologically realistic auditory stimulus. The RSS we used does produce noise as the spring uncoils, but it is not clear if the sound frequencies produced are similar to the sounds made by common single‐strike predators like snakes and owls. Kangaroo rats are especially sensitive to low‐frequency sounds associated with owl swoops and snake strikes, and it is possible that the frequencies of the sounds made by the RSS do not faithfully capture those frequencies. However, the only known differences in hearing ability between kangaroo rats and woodrats are that kangaroo rats have better low‐frequency hearing (i.e., woodrats do not have better high‐frequency hearing; Heffner & Heffner, 1985; Heffner & Masterton, 1980). Therefore, it is somewhat unlikely that the woodrats have faster reaction times because they are sensitive to sounds that the kangaroo rats cannot hear. Additionally, incorporating more precise measures of ambient light could help tease apart trends in rodent reaction time. While our measurements of ambient light were coarse and could not be included in statistical analyses, varying light level has been suggested to affect kangaroo rat escape success during predator–prey interactions by influencing their ability to see predators (Webster & Webster, 1971). Lastly, we acknowledge that auditory stimuli are not the only cue that the rodents may be picking up on, and other physiological mechanisms outside of sensory processing (e.g., the speed of muscle contraction impacted by dominant muscle fiber type) could factor into the interspecies differences in reaction time.

## 5 CONCLUSION

Although bipedalism is not necessary to be a good jumper, as in the case of the pocket mouse, it clearly provides an advantage when escaping from predators via jumping. Jumping reduces the amount of time needed to move out of a predator's trajectory (i.e., reduced take‐off time), and bipedal rodents were much more likely to jump as a general startle response than non‐heteromyid quadrupeds, which translates into better escape performance during attacks. When considering that pocket mice share important anatomical features with kangaroo rats, it is expected that their ability to jump would be more similar to these species than to other rodents. Therefore, we can conclude that relatively enlarged hindlimbs provide an advantage during predator escape maneuvers by increasing the ability to jump out of the attack trajectory, which is most exaggerated in bipedal rodents. These findings likely explain why heteromyids are more effective at evading predators (Kotler, 1985; Kotler et al., 1988; Longland & Price, 1991; Pierce et al., 1992). Future work could build on our foundation and directly examine the evolution of jumping performance and posture among a more diverse array of rodent species.

## AUTHOR CONTRIBUTIONS

**Grace A. Freymiller:** Conceptualization (equal); data curation (lead); formal analysis (lead); funding acquisition (equal); investigation (lead); methodology (equal); project administration (equal); resources (supporting); supervision (equal); validation (equal); visualization (lead); writing – original draft (lead); writing – review and editing (equal). **Malachi D. Whitford:** Conceptualization (equal); formal analysis (supporting); funding acquisition (supporting); investigation (equal); methodology (equal); project administration (equal); resources (supporting); supervision (equal); validation (equal); writing – original draft (supporting); writing – review and editing (equal). **Craig P. McGowan:** Conceptualization (equal); methodology (equal); resources (supporting); validation (equal); visualization (supporting); writing – review and editing (equal). **Timothy E. Higham:** Conceptualization (equal); funding acquisition (supporting); methodology (equal); resources (supporting); validation (equal); visualization (equal); writing – review and editing (equal). **Rulon W. Clark:** Conceptualization (equal); funding acquisition (equal); investigation (equal); methodology (equal); project administration (supporting); resources (lead); supervision (supporting); validation (equal); visualization (equal); writing – review and editing (equal).

## FUNDING INFORMATION

This project was funded by the American Society of Mammalogists (Grant‐in‐Aid of Research to GAF), American Philosophical Society (Lewis & Clark Fund for Field Exploration to GAF), Animal Behavior Society (Student Research Grant to MDW), the National Science Foundation (IOS‐1856404 to RWC and TEH), and San Diego State University (UGP 242557 to RWC and Graduate Student Travel Fund to GAF).

## CONFLICT OF INTEREST STATEMENT

The authors have no competing interests.

## Supporting information


Data S1.

## Data Availability

Data are accessible as a supplementary document, and supplementary videos are available at YouTube.com (see Table 1).
